# A global analysis of nasopharynx cancer burden attributable to occupational formaldehyde exposure

**DOI:** 10.3389/fonc.2025.1660669

**Published:** 2025-09-26

**Authors:** Jian Li, Yongjing Lai, Anzhou Tang, Xiang Yi

**Affiliations:** ^1^ Key Laboratory of Early Prevention and Treatment for Regional High Frequency Tumor (Guangxi Medical University), Ministry of Education, Nanning, China; ^2^ Department of Otorhinolaryngology Head and Neck Surgery, The First Affiliated Hospital of Guangxi Medical University, Nanning, China

**Keywords:** nasopharynx cancer, cancer burden, global burden of disease 2021, socio-demographic index, occupational formaldehyde exposure

## Abstract

**Background:**

Occupational formaldehyde is a significant risk factor for nasopharynx cancer. This study aimed to analyze the burden of nasopharynx cancer attributable to occupational formaldehyde using data from the Global Burden of Disease (GBD) 2021.

**Methods:**

Data on mortality and disability-adjusted life years (DALYs) rates were extracted from the GBD 2021. Joinpoint regression analysis was used to assess trends from 1990 to 2021. Health inequalities were evaluated using the slope index of inequality (SII) and concentration index (CI). An age-period-cohort (APC) model was employed to analyze independent effects, and the Bayesian age-period-cohort (BAPC) model was used to project the disease burden through 2036.

**Results:**

In 2021, nasopharynx cancer due to occupational formaldehyde exposure had 592.17 deaths (95% uncertainty interval (UI): 401.41 to 856.86) and 25,382.99 DALYs cases globally (95% UI: 16,268.27 to 36,800.17). The highest burden was observed in middle socio-demographic index (SDI) regions (254.25 deaths and 10,692.88 DALYs). East Asia had the highest burden (313.68 deaths and 13,030.97 DALYs cases), while Oceania had the lowest (0.33 deaths). From 1990 to 2021, global age-standardized mortality rates (ASMR) and age-standardized DALYs rates (ASDR) decreased, although significant increases were observed in the Caribbean and Central Asia. The highest burden was among males aged 45–49 years (88 deaths and 3,869 DALYs cases). Health inequality analysis showed a narrowing gap in DALY rates. A slight increase in nasopharynx cancer burden attributable to occupational formaldehyde exposure is projected by 2036, with a higher burden observed in males than in females.

**Conclusions:**

Despite an overall decline in the global burden of nasopharynx cancer attributable to occupational formaldehyde, significant regional disparities persist. Risks associated with gender and age highlight the necessity for enhanced occupational protection and targeted interventions to safeguard high-risk populations. Continued formaldehyde exposure monitoring remains crucial for informing and supporting global cancer prevention strategies.

## 1 Introduction

Nasopharynx cancer is an epithelial malignancy of the nasopharynx with a multifactorial etiology, marked by substantial heterogeneity in its genetic, viral, and environmental determinants (1). Epstein-Barr virus (EBV) infection is well recognized as a primary oncogenic driver, alongside genetic susceptibility and environmental exposures (2). In recent years, research on nasopharynx cancer has increasingly focused on its molecular pathological mechanisms, immune evasion strategies, and advancements in early diagnostic techniques (3, 4). Although improvements in diagnostic and therapeutic approaches have enhanced early detection rates and survival outcomes, nasopharynx cancer remains a significant global health concern due to its high mortality and disability-adjusted life years (DALYs) rates, particularly in regions with high incidence rates (5). While considerable research has elucidated the roles of viral and genetic risk factors, the contribution of environmental and occupational exposures, specifically formaldehyde, remains insufficiently explored (6).

Formaldehyde, a well-established occupational carcinogen, is a prevalent occupational hazard in various industries. Exposure to formaldehyde has been associated with multiple cancers, particularly in occupations such as construction, woodworking, and healthcare, where exposure levels are significantly high (7, 8). Epidemiological evidence indicates a significantly elevated risk of nasopharynx cancer among workers with prolonged formaldehyde exposure, particularly in high-exposure settings such as woodworking and pathology laboratories. The proposed carcinogenic mechanisms include DNA damage, oxidative stress, and chronic upper respiratory tract inflammation, which may promote tumorigenesis in the nasopharyngeal epithelium (9–11). However, comprehensive global assessments quantifying the disease burden attributable to formaldehyde exposure remain limited, particularly regarding regional and occupational variations in exposure levels.

The Global Burden of Disease (GBD) 2021 study provides a systematic framework for quantifying disease burden and risk factor contributions across populations. By integrating global estimates of mortality and DALYs rates, the GBD study enables a nuanced understanding of disease trends and health disparities (12). This study leverages GBD 2021 to assess the global burden of nasopharynx cancer attributable to occupational formaldehyde exposure from 1990 to 2021, with a focus on regional disparities between high, high-middle, middle, low-middle, and low socio-demographic index (SDI) regions. Temporal trends were examined using Joinpoint regression analysis, and Bayesian age-period-cohort (BAPC) modeling was employed to project future trends in burden. These findings from this analysis may be used to inform evidence-based policies and targeted interventions to mitigate occupational exposure risks and reduce disparities in the burden of nasopharynx cancer worldwide.

## 2 Methods

### 2.1 Data source

Data for this study were derived from the GBD 2021 study conducted by the Institute for Health Metrics and Evaluation (IHME) (https://ghdx.healthdata.org/gbd-2021) (13). This comprehensive analysis estimated mortality and DALYs rates associated with diseases, injuries, and risk factors across 204 countries and territories, organized into 21 GBD regions. Covering 371 diseases and injuries from 1990 to 2021, nasopharynx cancer is identified as a level 3 cause (ICD-10 code C11) (https://www.healthdata.org/research-analysis/diseases-injuries-risks/factsheets/2021-nasopharynx-cancer-level-3-disease) (14). All data utilized in this analysis are publicly accessible, negating the need for additional ethical approval. In GBD 2021, occupational exposure to formaldehyde was defined as employment in industrial sectors with exposure levels substantially exceeding background environmental concentrations. Classification followed the International Standard Industrial Classification (ISIC) codes, covering industries with established high exposure potential, including furniture and wood product manufacturing, chemical and formaldehyde production and use, and selected professional and healthcare occupations such as embalmers and pathologists. Exposure was modeled as a binary variable (exposed vs unexposed) based on sectoral employment. Consistent with its designation as a Group 1 carcinogen, the theoretical minimum risk exposure level (TMREL) for formaldehyde was assumed to be zero (https://www.healthdata.org/research-analysis/diseases). Complete avoidance of occupational formaldehyde exposure is thus necessary to achieve the lowest possible health risk.

### 2.2 Data collection

The DisMod-MR 2.1 framework was employed to model the GBD, integrating mortality data into a unified model to generate preliminary estimates for nasopharynx cancer mortality (15). DALYs, which combine years of life lost due to premature mortality and years lived with disability, were used as the primary metric to assess disease burden (16). To evaluate the impact of occupational exposure to formaldehyde, relevant data were retrieved from the GBD Results tool, selecting occupational exposure to formaldehyde as the risk factor and nasopharynx cancer as the outcome of interest (https://vizhub.healthdata.org/gbd-results).

The GBD 2021 study applied a comparative risk assessment framework to quantify disease burden attributable to occupational formaldehyde exposure. Theoretical minimum risk exposure level (TMREL) was defined as zero, consistent with its designation as a Group 1 carcinogen. Relative risks (RRs) for nasopharyngeal carcinoma were derived from systematic reviews and meta-analyses of epidemiological studies. Only estimates adjusted for confounders such as age, sex, smoking, and socioeconomic status were incorporated to reduce bias.

Exposure distributions were estimated using International Labour Organization records and occupational survey data, stratified by industry-specific probability and intensity of exposure. Population attributable fractions (PAFs) were calculated to reflect the proportion of nasopharyngeal carcinoma cases that could be prevented under elimination of exposure. Attributable deaths and disability-adjusted life years (DALYs) were estimated by multiplying PAFs with the total nasopharyngeal carcinoma burden in GBD 2021. To avoid double attribution, the exclusivity principle of GBD was applied. RRs for formaldehyde were obtained from studies controlling for co-exposures such as wood dust, ensuring that estimated burden reflected the independent effect of formaldehyde.

### 2.3 Statistical analysis

Joinpoint regression analysis was utilized to identify trends in the burden of nasopharynx cancer attributable to occupational formaldehyde from 1990 to 2021 (17). The analysis was performed using Joinpoint Regression Program (Version 5.1.0 Statistical Research and Applications Branch, National Cancer Institute, USA). We allowed a maximum of 5 joinpoints based on the default settings. The Monte Carlo permutation test with 4499 repetitions was used to identify the optimal number of joinpoints, with a significance level of 0.05. The annual percentage change (APC) and average annual percentage change (AAPC) were calculated for each segment and the overall period, respectively, along with their 95% confidence intervals (CI) (18, 19). Trends were defined as increasing if the APC or AAPC estimate and its 95% confidence interval (CI) lower bound were both greater than zero. Trends were considered decreasing if the APC or AAPC estimate and its 95% CI upper bound were both less than zero. Otherwise, trends were classified as stable.

The slope index of inequality (SII) and concentration index (CI) were used to quantify both absolute and relative inequalities in the burden of nasopharynx cancer across countries (20). Countries were ranked by SDI from lowest to highest. SII was estimated by fitting generalized linear regression models to quantify the association between national age-standardized mortality or DALYs rates and SDI rank. The index represents the predicted absolute difference in outcomes between hypothetical populations at the lowest (SDI = 0) and highest (SDI = 1) positions on the SDI scale. Negative values indicate concentration of adverse outcomes among lower-SDI populations, reflecting pro-poor inequality. Increasingly negative values over time signify widening absolute disparities (21). Relative inequality was assessed using the CI, derived from concentration curves plotting the cumulative distribution of health outcomes against the cumulative population share ranked by SDI. CI was defined as twice the area between the concentration curve and the equality line. Negative values indicate disproportionate concentration of disease burden in socioeconomically disadvantaged populations.

To predict future trends in ASMR and ASDR of nasopharynx cancer attributable to occupational formaldehyde, the Bayesian age-period-cohort (BAPC) model, combined with nested Laplace approximation, was used to project data through 2036 (22). The model’s goodness of fit was assessed via posterior predictive checks and residual analysis. Predictions were generated based on 1,000 posterior samples, with results expressed as median values alongside their 95% uncertainty intervals. The BAPC model was fitted to historical data using INLA, a technique that efficiently computes the posterior distributions of model parameters. Markov Chain Monte Carlo (MCMC) sampling was employed to derive the posterior distributions of these parameters, thereby generating a set of credible parameter values.

Trends in ASMR and ASDR attributable to occupational formaldehyde were assessed through calculation per 100,000 population using the following formula (23):


$$ASR=\frac{\sum_{i=1}^{A}a_{i}w_{i}}{\sum_{i=1}^{A}w_{i}}\times 100000$$

The EAPC was derived from a linear regression model fitted to the natural logarithm of the ASR from 1990 to 2021. In this formula, a_i_ represents the age-specific rate for the i^th^ age group, W_i_ indicates the population size of the i^th^ age group in the standardized population, and A denotes the total number of age groups.

estimated annual percentage change (EAPC) was derived from the regression model expressed as (24):


Y=α+βX+ϵ

Here, Y denotes the natural logarithm of the ASR (ASMR and ASDR for nasopharynx cancer attributable to occupational formaldehyde), X signifies the calendar year, α denotes the intercept, β represents the slope (indicating the trend), and ϵ is the error term.

The EAPC is computed using the formula:


EAPC=100×(exp(β)−1)

This computation reflects the annual percentage change, with the 95% CI for the EAPC established through linear regression analysis. The CI of EAPC is derived from the standard error of the regression slope β. An increasing trend is identified if both the EAPC and its 95% CI lower bound are positive. A decreasing trend is indicated when both the EAPC and its 95% CI upper bound are negative. Otherwise, the trend is considered stable.

Data analysis and extraction were performed using R software (version 4.2.1), with key packages including dplyr (version 1.1.4), viridis (version 0.6.5), mgcv (version 1.9—1), MASS (version 7.3—61), splines (version 4.4.1), tidyverse (version 2.0.0), BAPC (version 0.0.36), and INLA (version 23.06.29). Visualizations were generated using ggplot2. Joinpoint regression models were constructed using Joinpoint software (version 4.9.1.0; National Cancer Institute, Rockville, Maryland, USA) to identify significant trend changes from 1990 to 2021. The age-period-cohort (APC) models were employed to analyze the independent effects of age, period, and cohort on the burden of nasopharynx cancer attributable to occupational formaldehyde.

## 3 Results

### 3.1 Global trends

Globally, the burden of nasopharynx cancer attributable to occupational formaldehyde showed a declining trend from 1990 to 2021. In 2021, it led to an estimated 592.17 deaths (95% UI: 401.41 to 855.86) and 25,382.99 DALYs cases (95% UI: 16,268.27 to 36,800.17) globally. The burden of nasopharynx cancer attributable to occupational formaldehyde was most pronounced in the middle SDI regions, with 254.25 deaths (95% UI: 169.12 to 371.02) and 10,692.88 DALYs cases (95% UI: 6,904.89 to 15,403.19) (**Table 1**).

**Table 1:** Number of deaths, cases of DALYs, ASMR, and ASDR for nasopharynx cancer attributable to occupational formaldehyde in 2021.

Location	Deaths		DALYs	
	Number	ASMR (per 100,000 population)	Number	ASDR (per 100,000 population)
Global	592.17 (401.41,855.86)	0.007 (0.005,0.01)	25382.99 (16268.27,36800.17)	0.3 (0.193,0.435)
Low SDI	35.17 (22.07,53.56)	0.005 (0.003,0.007)	1653.51 (995.73,2515.49)	0.199 (0.124,0.306)
Low-middle SDI	103.1 (68.34,146.37)	0.006 (0.004,0.008)	4603.06 (2939.07,6526.06)	0.251 (0.161,0.356)
Middle SDI	254.25 (169.12,371.02)	0.009 (0.006,0.013)	10692.88 (6904.89,15403.19)	0.379 (0.246,0.541)
High-middle SDI	173.67 (113.19,266.7)	0.01 (0.006,0.015)	7391.76 (4617.84,11482.46)	0.449 (0.285,0.687)
High SDI	25.72 (17.1,37.18)	0.002 (0.001,0.003)	1030.57 (673.67,1505.33)	0.076 (0.048,0.109)
High-income	6.86 (4.7,9.59)	0 (0,0.001)	254.5 (179.74,352.68)	0.018 (0.013,0.025)
Australasia	0.16 (0.1,0.24)	0 (0,0.001)	6.62 (4.16,9.77)	0.018 (0.011,0.027)
Western Europe	2.43 (1.68,3.44)	0 (0,0.001)	90 (61.83,126.19)	0.016 (0.011,0.022)
High-income Asia Pacific	1.64 (1.08,2.38)	0.001 (0,0.001)	55.15 (37.29,78.28)	0.021 (0.015,0.03)
High-income North America	1.75 (1.23,2.5)	0 (0,0)	67.92 (46.77,95.68)	0.015 (0.01,0.021)
Southern Latin America	0.87 (0.58,1.27)	0.001 (0.001,0.002)	34.81 (22.93,50.73)	0.045 (0.03,0.066)
Central Europe, Eastern Europe, and Central Asia	4.52 (3.1,6.36)	0.001 (0.001,0.001)	194.88 (132.59,278.13)	0.04 (0.028,0.056)
Central Asia	2.33 (1.54,3.41)	0.002 (0.002,0.003)	107.35 (70.43,153.52)	0.106 (0.07,0.152)
Central Europe	1.03 (0.69,1.51)	0.001 (0,0.001)	39.66 (26.04,58.17)	0.026 (0.017,0.037)
Eastern Europe	1.16 (0.74,1.67)	0 (0,0.001)	47.87 (30.63,69.26)	0.018 (0.011,0.026)
Latin America and Caribbean	9.79 (6.81,13.3)	0.002 (0.001,0.002)	412.21 (283.33,548.11)	0.064 (0.044,0.084)
Central Latin America	3.34 (2.26,4.65)	0.001 (0.001,0.002)	138.78 (93.78,187.77)	0.052 (0.035,0.07)
Caribbean	1.59 (1.08,2.32)	0.003 (0.002,0.004)	63.81 (42.84,90.82)	0.123 (0.083,0.174)
Tropical Latin America	4.31 (2.93,5.99)	0.002 (0.001,0.002)	187.1 (126.38,255.39)	0.072 (0.048,0.097)
Andean Latin America	0.55 (0.36,0.76)	0.001 (0.001,0.001)	22.52 (14.62,31.15)	0.034 (0.022,0.047)
North Africa and Middle East	17.55 (11.23,25.56)	0.003 (0.002,0.004)	781.02 (488.4,1155.53)	0.122 (0.078,0.177)
South Asia	105.5 (68.65,154.34)	0.006 (0.004,0.009)	4710 (2946.03,6878.06)	0.254 (0.163,0.371)
Southeast Asia, East Asia, and Oceania	412.85 (273.2,624.42)	0.015 (0.01,0.022)	17346.5 (11213.51,25698.15)	0.646 (0.41,0.957)
East Asia	313.68 (199.88,488.32)	0.016 (0.01,0.024)	13030.97 (8173.36,20336.82)	0.688 (0.433,1.047)
Southeast Asia	98.84 (65.11,138.34)	0.013 (0.008,0.018)	4301.25 (2797.14,6114.94)	0.553 (0.36,0.781)
Oceania	0.33 (0.18,0.53)	0.003 (0.002,0.005)	14.29 (7.99,23.61)	0.121 (0.069,0.198)
Sub-Saharan Africa	35.1 (21.36,52.37)	0.004 (0.003,0.007)	1683.88 (1028.9,2549.57)	0.2 (0.121,0.299)
Southern Sub-Saharan Africa	1.17 (0.78,1.62)	0.001 (0.001,0.002)	56.22 (35.59,78.7)	0.069 (0.045,0.097)
Eastern Sub-Saharan Africa	24.42 (14.99,37.94)	0.009 (0.005,0.013)	1171.36 (707.82,1848.18)	0.38 (0.233,0.586)
Central Sub-Saharan Africa	1.31 (0.75,2.09)	0.001 (0.001,0.002)	60.97 (35.4,97.34)	0.062 (0.035,0.099)
Western Sub-Saharan Africa	8.2 (4.69,12.7)	0.002 (0.001,0.004)	395.33 (220.93,620.26)	0.113 (0.064,0.176)

DALYs, disability-adjusted life years; ASMR, age-standardized mortality rates; ASDR, age-standardized DALYs rates.

### 3.2 Regional trends

**Figure 1:**
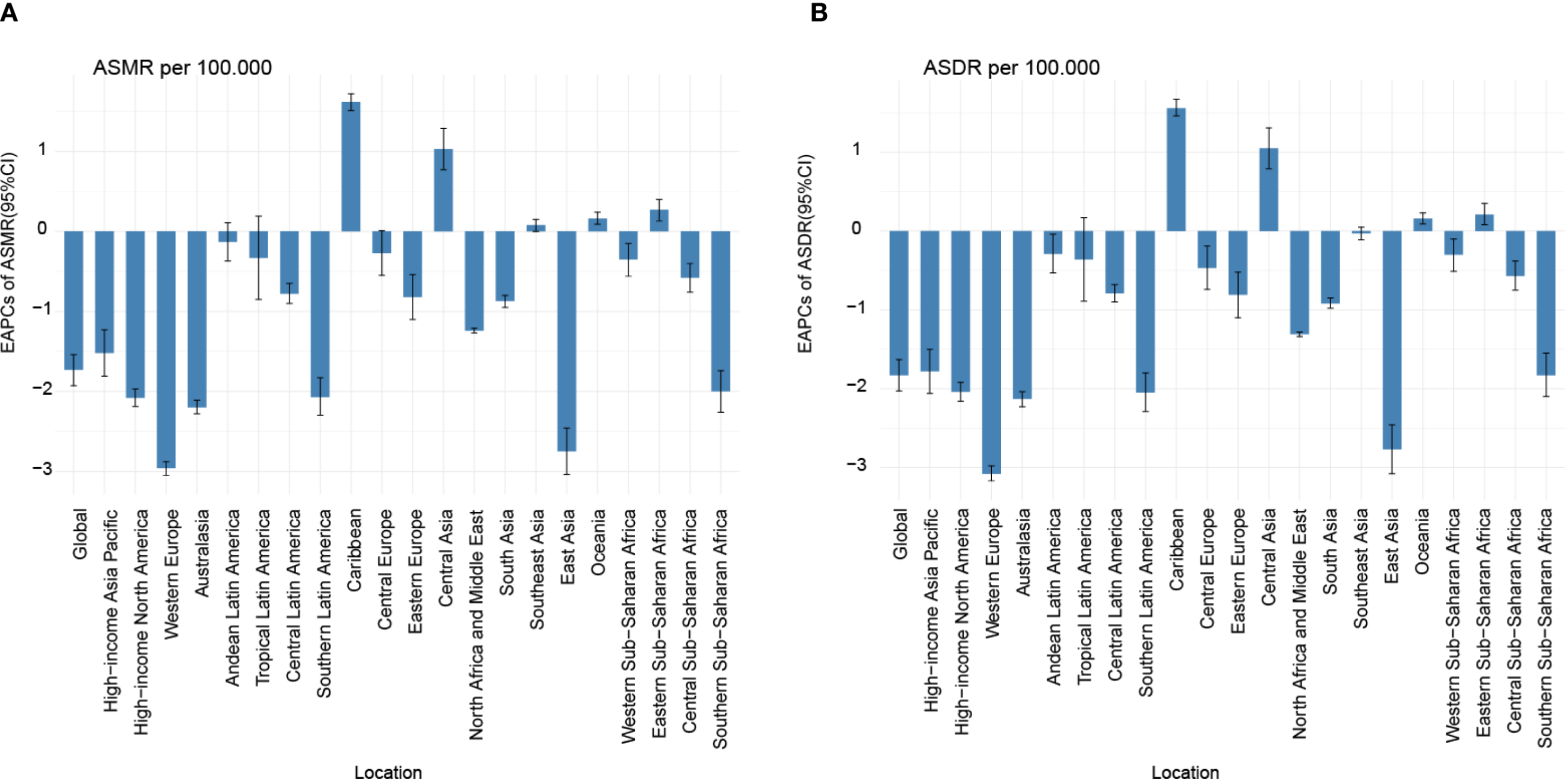
EAPC of ASMR **(A)** and ASDR **(B)** for nasopharynx cancer attributable to occupational formaldehyde for both sexes from 1990 to 2021. EAPC, estimated annual percentage change.

Substantial disparities in disease burden were observed across GBD regions. Among the 21 GBD regions, East Asia had the highest burden, with 313.68 deaths (95% UI: 199.88 to 488.32) and 13,030.97 DALYs cases (95% UI: 8,173.36 to 20,336.82), while Australasia had the lowest burden, with 0.16 deaths (95% UI: 0.10 to 0.24) and 6.62 DALYs cases (95% UI: 4.16 to 9.77). From 1990 to 2021, the EAPC in ASMR and ASDR for nasopharynx cancer attributable to occupational formaldehyde showed a general decline across most regions. Notably, East Asia and Western Europe experienced significant reductions, with EAPC of -2.75 (95% CI: -3.04 to -2.46) and -2.96 (95% CI: -3.05 to -2.88) for ASMR, and -2.77 (95% CI: -3.08 to -2.46) and -3.08 (95% CI: -3.17 to -2.98) for ASDR. In contrast, the Caribbean and Central Asia had substantial increases in both ASMR and ASDR, with EAPC of 1.62 (95% CI: 1.51 to 1.72) and 1.03 (95% CI: 0.77 to 1.29) for ASMR, and 1.56 (95% CI: 1.46 to 1.67) and 1.05 (95% CI: 0.79 to 1.31) for ASDR (**Figure 1**).

### 3.3 National trends

**Figure 2:**
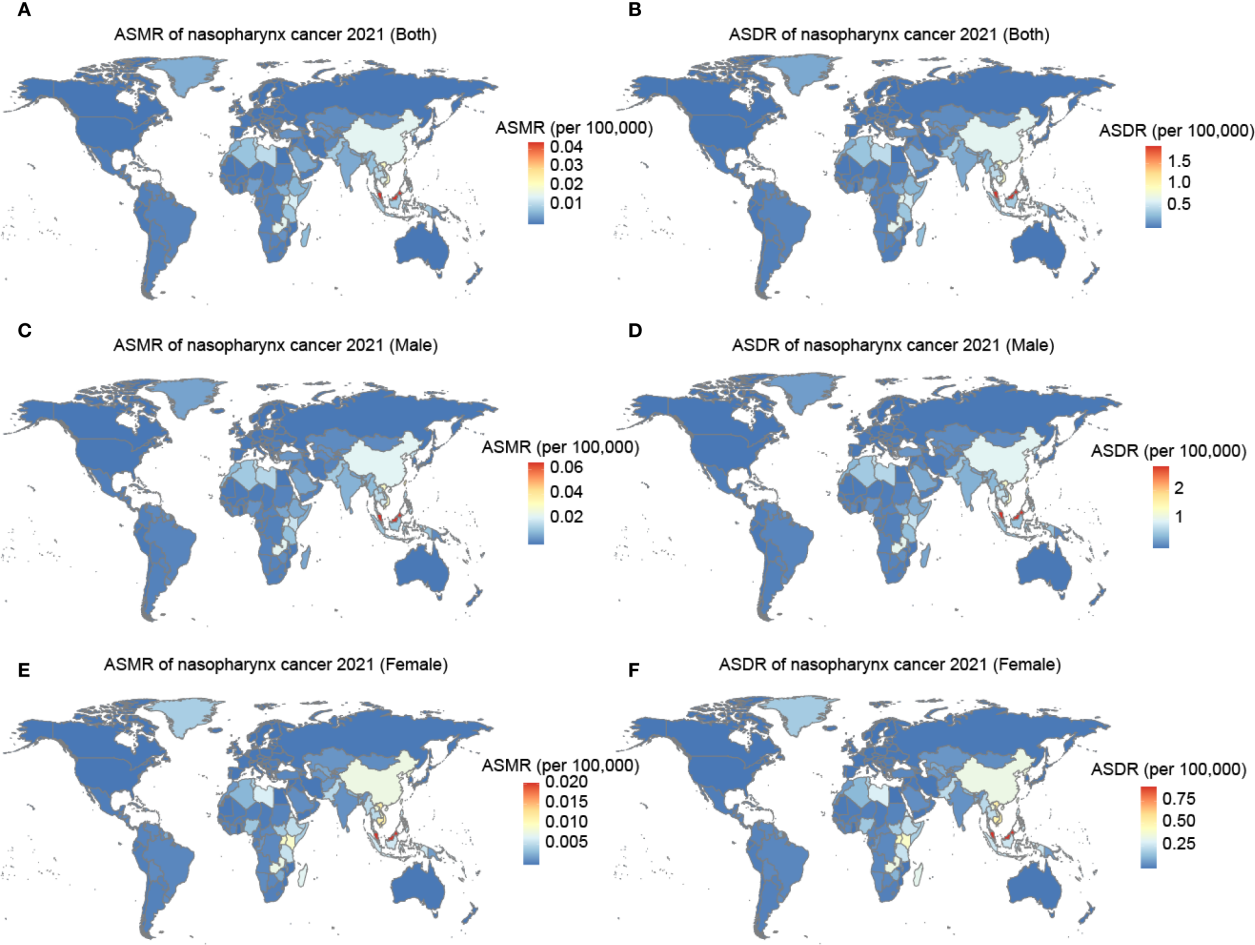
The ASMR and ASDR for nasopharynx cancer attributable to occupational formaldehyde in 204 countries and territories in 2021. **(A)** ASMR of both. **(B)** ASDR of both. **(C)** ASMR of male. **(D)** ASDR of male. **(E)** ASMR of female. **(F)** ASDR of female.

At the national level, the burden was generally low but highly variable. Among 204 countries and territories, the burden of nasopharynx cancer due to occupational formaldehyde was generally low. However, countries such as Malaysia, Taiwan (China), and Vietnam had relatively high burdens. These regions had ASMR of 0.044 (95% UI: 0.03 to 0.07), 0.023 (95% UI: 0.01 to 0.03), and 0.021 (95% UI: 0.01 to 0.03), respectively, and ASDR of 1.93 (95% UI: 1.17 to 2.86), 0.98 (95% UI: 0.60 to 1.47), and 0.907 (95% UI: 0.54 to 1.40) (**Figure 2**, **Supplementary Table S1**). In China, the burden of nasopharynx cancer attributable to occupational formaldehyde exposure was relatively high among males and females in 2021, with ASMR of 0.02 (95% UI: 0.01, 0.03) and 0.01 (95% UI: 0.005, 0.01), respectively.

### 3.4 Joinpoint regression analysis

**Figure 3:**
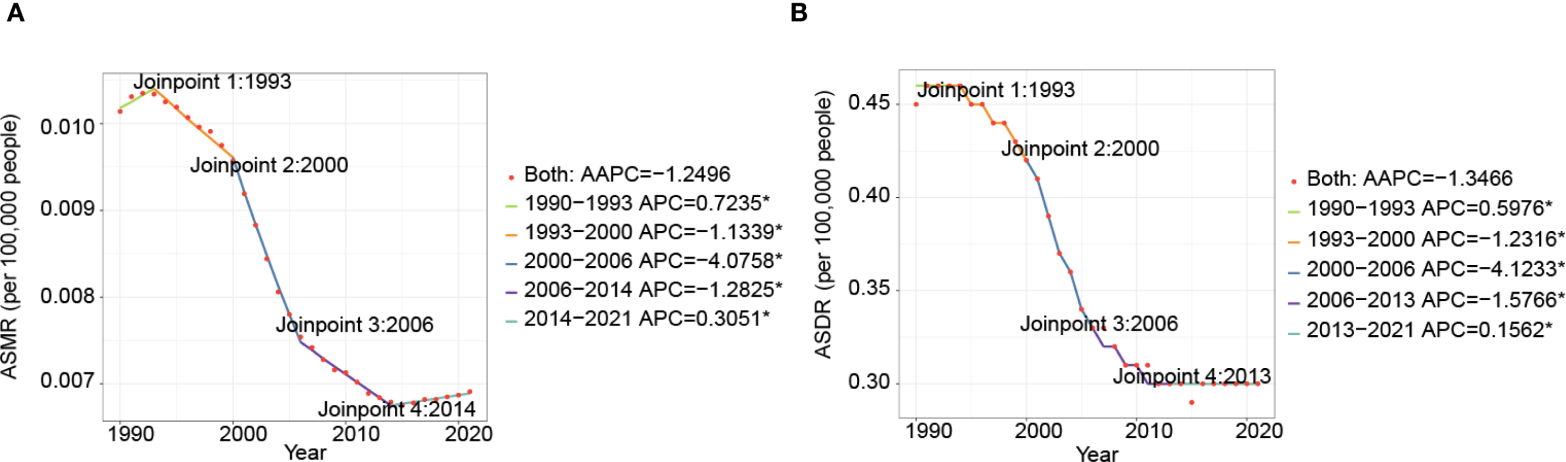
Time trends of ASMR **(A)**, and ASDR **(B)** for nasopharynx cancer attributable to occupational formaldehyde for both sexes from 1990 to 2021.

Globally, both ASMR and ASDR for nasopharynx cancer attributed to occupational formaldehyde showed a declining trend from 1990 to 2021 (AAPC of ASMR: -1.2496, AAPC of ASDR: -1.3466). Joinpoint regression analysis identified significant variations in APC across different periods (**Figure 3**, **Supplementary Table S2**). For example, ASMR increased by 0.7325 (95% CI: 0.2155 to 1.2341) from 1990 to 1993 and by 0.3051 (95% CI: 0.1637 to 0.4468) from 2014 to 2021, while ASDR increased by 0.5976 (95% CI: 0.0722 to 1.1258) and 0.1562 (95% CI: 0.0379 to 0.2746), respectively.

### 3.5 The burden of nasopharynx cancer attributable to occupational formaldehyde by gender and age

**Figure 4:**
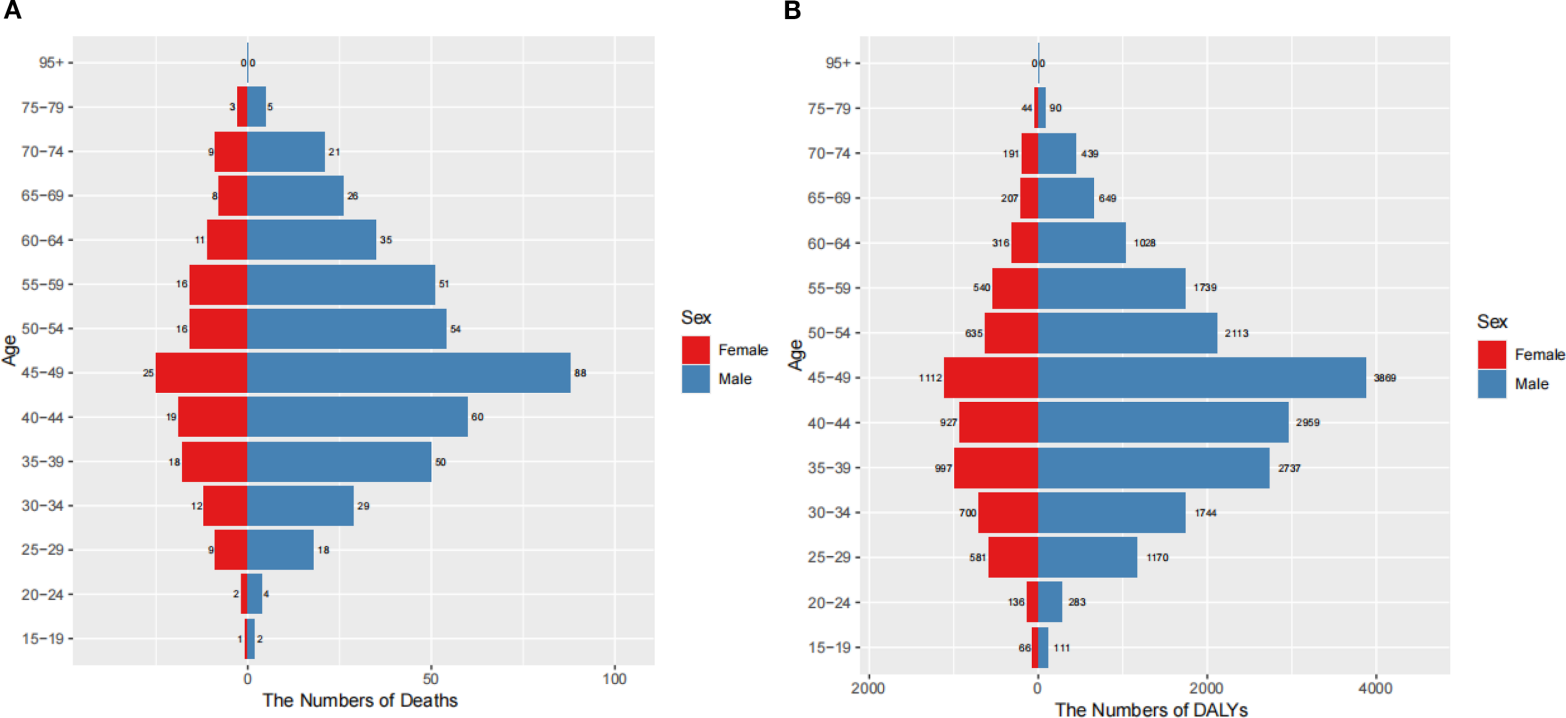
Burden of nasopharynx cancer attributable to occupational formaldehyde by age and gender in 2021. **(A)** The numbers of deaths. **(B)** The numbers of DALYs.

Marked gender and age disparities were observed in the burden of nasopharyngeal cancer attributable to occupational formaldehyde. In 2021, significant gender and age disparities were observed in the burden of nasopharyngeal cancer attributable to occupational formaldehyde (**Figure 4**). The number of deaths and DALYs increased with age, peaking in the 45–49 years age group, where males experienced 88 deaths and 3,869 DALYs cases, while females had 25 deaths and 1,112 DALYs cases. The burden declined in older age groups, with males generally having a higher burden than females, particularly in the 45–49 age group.

### 3.6 Health inequality analysis

**Figure 5:**
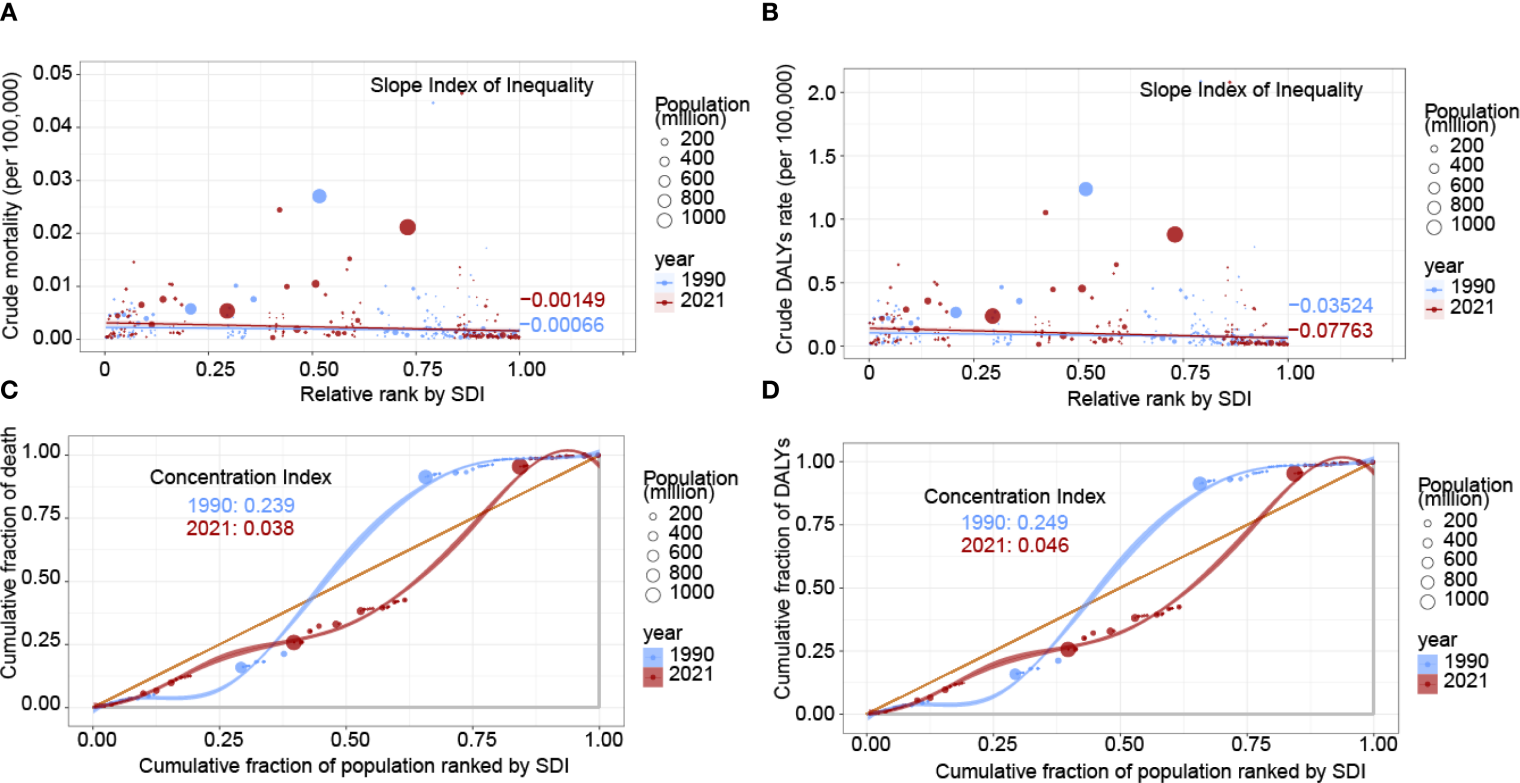
Health inequality regression and concentration curves for the burden of nasopharynx cancer attributable to occupational formaldehyde in 1990 and 2021. **(A)** SII for mortality. **(B)** SII for DALYs rates. **(C)** CI for mortality. **(D)** CI for DALYs rates. Scatter plots represent countries and regions categorized by population size. A and B depict the relationship between mortality and DALYs rates attributable to occupational formaldehyde exposure and the SDI. C and D show the CI, which quantifies relative inequality by measuring the area under the Lorenz curve, aligning the distribution of deaths and DALYs with the population distribution by SDI.

From 1990 to 2021, the SII for mortality decreased from -0.00066 to -0.00149, the SII for DALYs rates worsened, shifting from -0.03524 in 1990 to -0.07763 in 2021, suggesting an increase in health inequality. The contrasting trends in the SII for mortality and DALYs rates highlight a rising disease burden of nasopharyngeal cancer attributable to occupational formaldehyde exposure with increasing SDI, implying higher health indicator values in lower SDI regions. Additionally, the CI for mortality declined from 0.239 in 1990 to 0.038 in 2021, and the CI for DALYs rates decreased from 0.249 to 0.046, both indicating a reduction in health inequality (**Figure 5**). The coexistence of increasingly negative SII values with declining CI estimates reflects the classical “absolute increase-relative decrease” paradox. This pattern arises when overall burden declines across all SDI groups, but reductions occur more rapidly in higher-SDI populations, thereby widening absolute gaps while narrowing relative disparities.

### 3.7 APC model analysis

**Figure 6:**
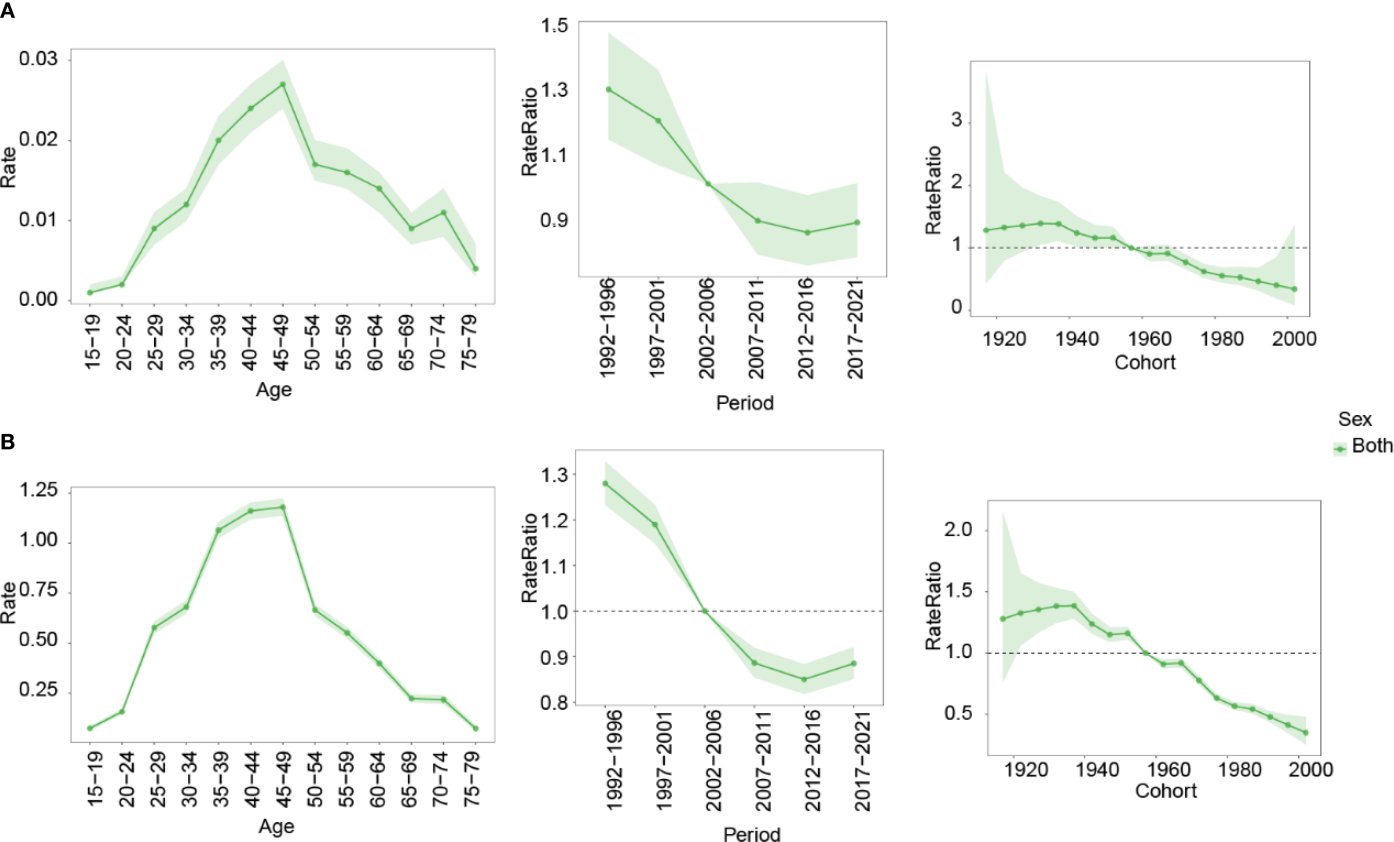
Impact of age, period, and birth cohort on the RR of nasopharynx cancer attributable to occupational formaldehyde for both sexes. **(A)** mortality. **(B)** DALYs rates. RR, risk ratio.

The APC modeling revealed distinct patterns in the burden of nasopharynx cancer attributable to occupational formaldehyde across different ages, periods, and birth cohorts (**Figure 6**). Age effects indicated that mortality and DALYs rates increased with age, peaking in the 45–49 years age group before declining. Period effects showed a continuous decrease in risk ratios (RR), and DALY rates exhibited a continuous downward trend from 1992 to 1996 and from 2012 to 2016. The highest RR was observed from 1992 to 1996, with values of 1.285 (95% CI: 1.034-1.598) for mortality and 1.275 (95% CI: 0.949-1.712) for DALY rates, respectively. The lowest RR were recorded in 2012-2016, with values of 0.853 (95% CI: 0.684-1.064) for mortality and 0.853 (95% CI: 0.635-1.147) for DALYs rates. Cohort effects revealed the highest RR for mortality and DALY rates in the 1895–1899 birth cohort (2.812 and 3.779, respectively), and the lowest in the 2000–2004 cohort (0.294 and 0.303).

### 3.8 Future prediction

**Figure 7:**
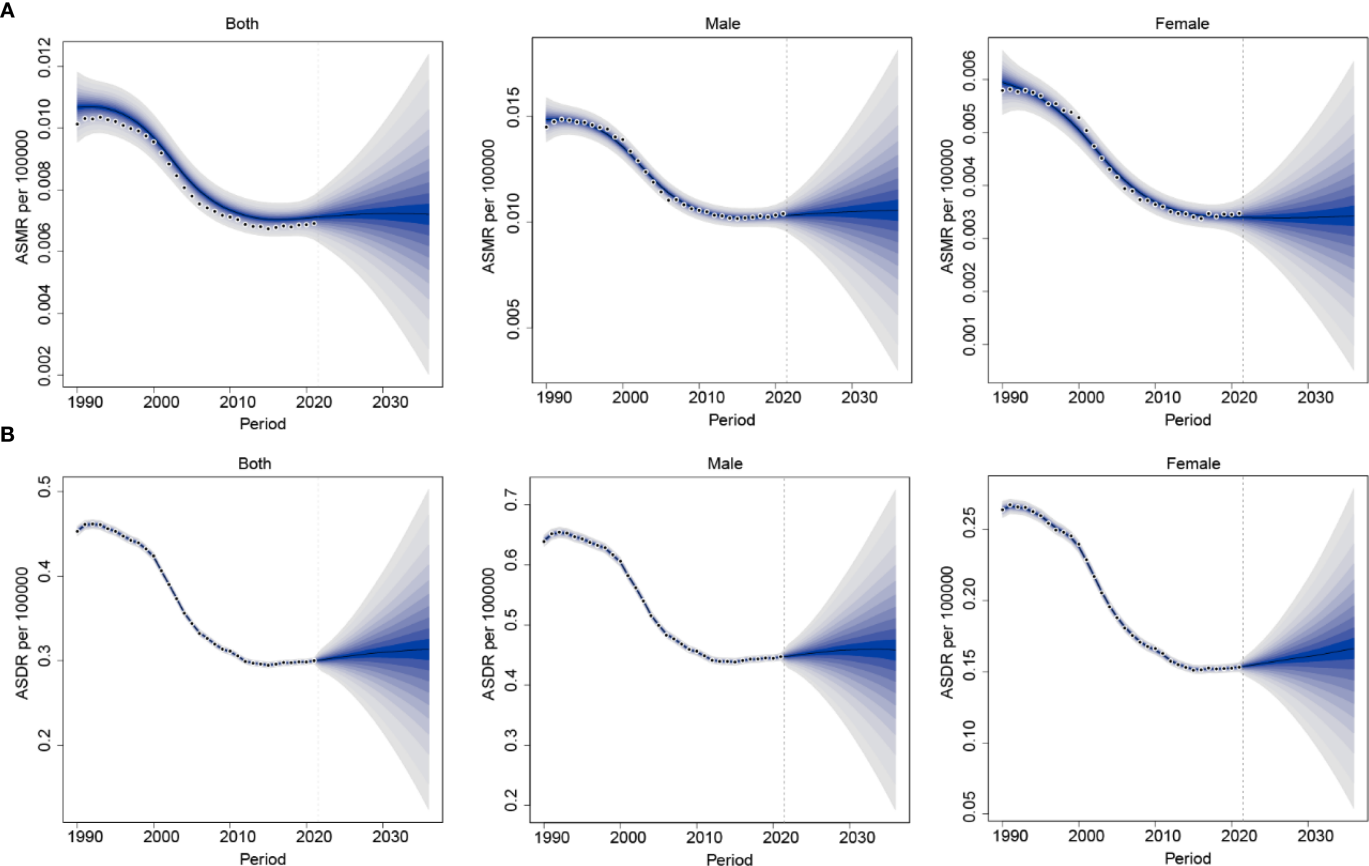
Future projections of the global burden of nasopharynx cancer attributable to occupational formaldehyde. **(A)** ASMR. **(B)** ASDR.

The BAPC model was used to project the future burden of nasopharynx cancer attributable to occupational formaldehyde from 2022 to 2036. Projections indicate a slight increase in the burden of nasopharynx cancer attributable to occupational formaldehyde exposure by 2036. ASMR and ASDR for both sexes are estimated to change from 0.0071 (95% CI: 0.006–0.008) and 0.301 (95% CI: 0.288–0.314) in 2022 to 0.0034 (95% CI: 0.0005–0.0063) and 0.313 (95% CI: 0.124–0.502) in 2036, respectively. A consistently higher disease burden is projected among males compared with females, the ASDR is estimated at 0.166 (95% CI: 0.054–0.279) in males, whereas the corresponding value in females is 0.147 (95% CI: 0.054–0.279) by 2036 (**Figure 7**).

## 4 Discussion

This study, based on GBD 2021, provides a comprehensive assessment of the global burden of nasopharynx cancer attributable to occupational formaldehyde exposure, highlighting three key findings. First, the global ASMR and ASDR have exhibited a consistent decline (AAPC of -0.28 and -0.32, respectively), yet marked regional disparities persist. Second, a nonlinear association was observed between the SDI and disease burden, with middle SDI regions experiencing the highest disease burden. Third, an “absolute disparity widening-relative disparity narrowing” paradox was identified, underscoring complex inequities in occupational health.

Between 1990 and 2021, nasopharynx cancer mortality and DALYs rates for nasopharynx cancer attributable to occupational formaldehyde exposure declined globally, likely reflecting improvements in occupational safety regulations, early detection, and advances in therapeutic interventions, including precision immunotherapies (25–27). However, these global trends mask substantial geographic heterogeneity. East Asia had the highest absolute burden (313.68 deaths, 13,030.97 DALYs cases), yet demonstrated significant reductions in ASMR (EAPC: -2.75). Conversely, the Caribbean and Central Asia exhibited rising trends in ASMR (EAPC of 1.62 and 1.03, respectively), highlighting disparities in occupational health policies and their implementation. Notably, projections indicate an increasing burden among females, emphasizing the need for further investigation and tailored preventive strategies.

Nasopharynx cancer exhibits distinct geographical patterns, predominantly affecting East and Southeast Asia (2, 28). Consistent with prior research, our findings indicate that middle-SDI regions, including China, Malaysia, Taiwan (Provinces of China), and Vietnam, experience the highest burden, likely due to a confluence of genetic predisposition, environmental exposures, and occupational risk factors. A study utilizing GBD 2019 data similarly identified this geographic heterogeneity, suggesting that genetic, racial, environmental, and lifestyle factors contribute to the observed disparities (29).

While overall trends suggest a global decline, the increasing burden in low SDI regions, particularly in the Caribbean and Central Asia, raises concerns. This pattern aligns with previous findings demonstrating substantial variation in occupational health policy efficacy (30). High SDI countries, such as the United States and Western European nations, have implemented stringent regulations to minimize occupational formaldehyde exposure, contributing to declining incidence and mortality rates. In contrast, low SDI regions often face inadequate occupational health regulations, weak enforcement, and delayed adoption of protective measures, leading to pronounced spatial and temporal heterogeneity in formaldehyde exposure.

This study employed the SII and CI to quantify health inequities in nasopharynx cancer. From 1990 to 2021, absolute disparities in mortality and DALYs rates widened by 125% and 120%, respectively, highlighting an increasing divide between high and low SDI regions. This disparity may be attributed to the uneven distribution of advanced treatments, such as PD-1 inhibitors, which remain largely inaccessible in low-resource settings (31).

Paradoxically, the decline in relative inequality (as reflected in CI reductions for mortality: -84% and DALYs rates: -82%) suggests an overall improvement in health equity, potentially driven by enhanced occupational safety regulations in middle SDI countries. This complex interplay between economic development and health disparities underscores the need for targeted policy interventions that prioritize both regulatory enforcement and equitable access to preventive and therapeutic resources.

The demographic analysis of nasopharyngeal cancer associated with occupational formaldehyde exposure reveals marked gender and age disparities. Our findings indicate that men are at a significantly higher risk than women, particularly within the 45–49 age cohort, which bears the greatest burden of disease. In this demographic, male fatalities reached 88 deaths, translating to 3,869 DALYs cases, while female deaths totaled 25, corresponding to 1,112 DALYs cases. These statistics align with the documented male-to-female incidence ratio of approximately 2.2-3:1, highlighting the need for further exploration of underlying factors (32). Several factors contribute to these disparities. First, the occupational landscape exhibits a gender bias, with male-dominated fields, such as construction and wood processing, posing higher risks for formaldehyde exposure. Second, protective behavior differences emerge, as male workers are found to be less likely to utilize protective equipment, including masks and goggles, compared to their female counterparts, which heightens their exposure risk. Additionally, biological considerations may also play a critical role; epidemiological evidence suggests that estrogen may confer some protective advantages against nasopharyngeal cancer, potentially supporting an increased vulnerability among men under similar exposure conditions (32–34).

The peak incidence observed in the 45–49 age group can be explained through the lens of cumulative exposure. Formaldehyde, classified as a Group 1 carcinogen, demonstrates a well-established correlation between carcinogenic effects and exposure levels. Workers in this age bracket frequently experience 20–25 years of exposure, resulting in an accumulation that may surpass established carcinogenic thresholds (35–37). Furthermore, immune senescence associated with aging may impair the body’s capacity to eliminate abnormal cells, thereby facilitating carcinogenesis through synergistic mechanisms.

Projections indicate a slight increase in the burden of nasopharynx cancer attributable to occupational formaldehyde exposure by 2036. A higher burden is consistently observed among males, whereas females show a relative increase in disease impact, reflecting evolving occupational exposure patterns. These gender-specific trends likely result from differences in occupational exposure and workplace safety improvements. In male-dominated industries, such as woodworking and automobile manufacturing, automation, closed systems, enhanced ventilation, and substitution of formaldehyde-containing products have contributed to controlled exposure levels, yet the overall burden remains higher in males due to historical exposure patterns (34, 38). Conversely, female employment has increased in sectors such as cosmetics manufacturing and nail services, where outdated monitoring and limited protective measures sustain modest exposure risk, contributing to a gradual rise in disease burden (39, 40). These findings underscore the importance of tailored, region-specific occupational health strategies. High SDI regions should prioritize technology transfer and collaborative oncology platforms to reduce treatment inequities, middle SDI regions need enhanced monitoring and optimized protective equipment distribution, particularly in informal sectors, and low SDI regions require basic health monitoring systems and locally adapted exposure standards. Screening programs targeting high-risk populations, combined with gender-specific protective measures and health education, could mitigate the projected disease burden.

A large-scale occupational exposure study by the National Cancer Institute (NCI) confirmed a significant association between formaldehyde exposure and the risk of nasopharyngeal cancer (41). In light of this evidence, the International Agency for Research on Cancer (IARC) has classified formaldehyde as a human carcinogen associated with both nasopharyngeal cancer and leukemia (42). While the link between formaldehyde exposure and carcinogenesis is well-established through experimental and epidemiological research, the mechanistic pathways, particularly the key regulatory nodes in the molecular processes, require further investigation. Future research should focus on multi-omics integration models to unravel the synergistic effects of genotoxic and non-genotoxic mechanisms. Establishing exposure threshold functions through well-designed prospective cohort studies to refine the biological foundations underlying occupational exposure limits. Additionally, creating three-dimensional organoid models that can dynamically replicate the malignant transformations prompted by formaldehyde exposure. These advancements will provide critical insights necessary for formulating region-specific protective strategies, especially in industrial areas characterized by high exposure levels and limited safeguards in lower-SDI countries.

The implications of this study are profound for global cancer prevention and control strategies. Enhancing occupational health policies, particularly those aimed at protecting high-risk populations, can substantially reduce the burden of nasopharyngeal cancer attributable to formaldehyde exposure, thereby promoting health equity worldwide. International organizations and high-income nations should play a pivotal role in assisting lower- and middle-income regions by facilitating technology transfer and providing resources to bolster occupational health monitoring and intervention efforts, ultimately advancing global health objectives. However, this study is not without limitations. First, the quality and reliability of occupational exposure may vary due to the data extraction methods employed in the GBD 2021 study, potentially leading to an underrepresentation of informal employment. Second, although the GBD framework incorporated adjusted relative risks and applied the principle of risk attribution exclusivity to isolate the effect of occupational formaldehyde, residual confounding cannot be fully excluded. For example, if wood dust exposure in the source studies used to derive risk estimates was not adequately measured or adjusted for, part of the effect attributed to formaldehyde may, in fact, reflect wood dust exposure. Similarly, complex interactions between formaldehyde and other factors, such as Epstein-Barr virus infection, were not explicitly modeled within the GBD framework. Such limitations may bias estimates upward, as relative risks could capture effects of correlated or imperfectly measured co-exposures rather than formaldehyde alone. Lastly, the assessment of occupational exposure primarily relies on job classification rather than individual exposure data, which may introduce misclassification risks in exposure estimation.

## 5 Conclusion

This investigation delineates the global burden and evolving trends of nasopharyngeal cancer attributable to occupational formaldehyde exposure through a comprehensive multidimensional analysis. While there is an overall improvement in global trends, significant regional disparities, gender imbalances, and socio-economic development inequalities persist, contributing to notable health gaps. The “absolute gap widening-relative gap narrowing” health equity paradox highlighted in this study offers a fresh perspective on the intricate relationship between socio-economic development and health equity. Informed by our findings, tiered intervention strategies should be designed to concentrate on high-risk populations and regions, reinforcing occupational exposure monitoring and safety standards, particularly in low- and middle-income countries. Future research endeavors should aim to elucidate the molecular mechanisms underpinning formaldehyde-induced carcinogenesis, establish safe exposure thresholds, and assess the efficacy of various interventions, thereby providing a robust scientific foundation for the development of global occupational health policies.

## Data Availability

Publicly available datasets were analyzed in this study. This data can be found here: https://vizhub.healthdata.org/gbd-results/.
