# Supplementary material for: A global analysis of nasopharynx cancer burden attributable to occupational formaldehyde exposure
Source: Front Oncol. 2025 Sep 26;15:1660669. doi: 10.3389/fonc.2025.1660669 (PMC12510838; doi:10.3389/fonc.2025.1660669)
Supplement: Supplementary file 1 [file Table1.docx]

Table S1 The number of deaths, number of DALYs, ASMR, and ASDR for nasopharynx cancer attributable to occupational formaldehyde in 204 countries and territories in 2021.

|  | Deaths | | DALYs | |
| --- | --- | --- | --- | --- |
| Location | Number | Age-standardized rate(per 100,000 population) | Number | Age-standardized rate(per 100,000 population) |
| China | 301.058 | 0.016 | 12511.645 | 0.684 |
| Taiwan (Province of China) | 8.052 | 0.023 | 193.883 | 0.980 |
| Cambodia | 2.483 | 0.016 | 325.437 | 0.663 |
| Democratic People's Republic of Korea | 4.572 | 0.014 | 109.217 | 0.588 |
| Lao People's Democratic Republic | 0.724 | 0.011 | 32.938 | 0.472 |
| Maldives | 0.007 | 0.001 | 1262.819 | 0.047 |
| Philippines | 11.260 | 0.011 | 661.627 | 0.462 |
| Indonesia | 29.182 | 0.009 | 0.308 | 0.399 |
| Malaysia | 14.781 | 0.044 | 187.459 | 1.930 |
| Myanmar | 4.326 | 0.008 | 46.194 | 0.324 |
| Thailand | 10.132 | 0.011 | 504.596 | 0.501 |
| Sri Lanka | 1.128 | 0.004 | 427.844 | 0.184 |
| Timor-Leste | 0.074 | 0.007 | 3.242 | 0.309 |
| Kiribati | 0.003 | 0.003 | 1054.886 | 0.122 |
| Viet Nam | 24.503 | 0.021 | 0.453 | 0.907 |
| Fiji | 0.011 | 0.001 | 0.129 | 0.048 |
| Micronesia (Federated States of) | 0.004 | 0.005 | 0.083 | 0.190 |
| Marshall Islands | 0.002 | 0.004 | 0.187 | 0.152 |
| Samoa | 0.011 | 0.006 | 9.127 | 0.272 |
| Papua New Guinea | 0.207 | 0.003 | 1.683 | 0.106 |
| Tonga | 0.002 | 0.003 | 0.101 | 0.114 |
| Solomon Islands | 0.038 | 0.007 | 0.485 | 0.302 |
| Vanuatu | 0.010 | 0.004 | 0.418 | 0.163 |
| Armenia | 0.046 | 0.001 | 1.856 | 0.052 |
| Azerbaijan | 0.085 | 0.001 | 3.772 | 0.030 |
| Kazakhstan | 0.488 | 0.002 | 4.021 | 0.106 |
| Georgia | 0.098 | 0.002 | 3.338 | 0.097 |
| Mongolia | 0.072 | 0.002 | 21.660 | 0.096 |
| Kyrgyzstan | 0.168 | 0.003 | 7.933 | 0.118 |
| Turkmenistan | 0.124 | 0.002 | 6.209 | 0.111 |
| Tajikistan | 0.128 | 0.001 | 5.893 | 0.065 |
| Uzbekistan | 1.118 | 0.003 | 52.670 | 0.146 |
| Bosnia and Herzegovina | 0.008 | 0.000 | 0.304 | 0.007 |
| Albania | 0.013 | 0.000 | 2.434 | 0.016 |
| Croatia | 0.023 | 0.000 | 0.495 | 0.015 |
| Bulgaria | 0.064 | 0.001 | 0.839 | 0.027 |
| Hungary | 0.124 | 0.001 | 0.428 | 0.036 |
| Czechia | 0.071 | 0.000 | 2.667 | 0.019 |
| Montenegro | 0.001 | 0.000 | 4.647 | 0.005 |
| North Macedonia | 0.011 | 0.000 | 0.037 | 0.014 |
| Poland | 0.283 | 0.001 | 10.878 | 0.021 |
| Serbia | 0.055 | 0.000 | 2.459 | 0.018 |
| Romania | 0.297 | 0.001 | 11.552 | 0.046 |
| Slovenia | 0.007 | 0.000 | 2.071 | 0.010 |
| Belarus | 0.053 | 0.000 | 0.272 | 0.017 |
| Slovakia | 0.063 | 0.001 | 2.124 | 0.034 |
| Latvia | 0.009 | 0.000 | 0.224 | 0.014 |
| Estonia | 0.006 | 0.000 | 0.501 | 0.014 |
| Republic of Moldova | 0.024 | 0.000 | 0.929 | 0.019 |
| Lithuania | 0.013 | 0.000 | 0.334 | 0.015 |
| Ukraine | 0.283 | 0.000 | 11.855 | 0.021 |
| Russian Federation | 0.774 | 0.000 | 31.900 | 0.017 |
| Brunei Darussalam | 0.017 | 0.003 | 0.722 | 0.135 |
| Republic of Korea | 0.341 | 0.000 | 31.592 | 0.017 |
| Japan | 1.019 | 0.000 | 5.716 | 0.017 |
| Singapore | 0.022 | 0.003 | 12.772 | 0.125 |
| New Zealand | 0.268 | 0.000 | 10.067 | 0.015 |
| Australia | 0.142 | 0.000 | 0.902 | 0.018 |
| Andorra | 0.000 | 0.000 | 2.584 | 0.010 |
| Belgium | 0.070 | 0.000 | 0.013 | 0.018 |
| Denmark | 0.017 | 0.000 | 0.122 | 0.008 |
| France | 0.483 | 0.001 | 1.452 | 0.022 |
| Austria | 0.040 | 0.000 | 0.599 | 0.012 |
| Cyprus | 0.003 | 0.000 | 15.367 | 0.007 |
| Finland | 0.011 | 0.000 | 0.385 | 0.006 |
| Greece | 0.067 | 0.000 | 2.463 | 0.018 |
| Germany | 0.432 | 0.000 | 18.239 | 0.013 |
| Iceland | 0.002 | 0.000 | 0.064 | 0.015 |
| Israel | 0.036 | 0.000 | 13.046 | 0.014 |
| Luxembourg | 0.003 | 0.000 | 0.645 | 0.011 |
| Ireland | 0.017 | 0.000 | 0.101 | 0.010 |
| Italy | 0.353 | 0.000 | 1.402 | 0.016 |
| Netherlands | 0.098 | 0.000 | 0.279 | 0.016 |
| Portugal | 0.098 | 0.001 | 3.531 | 0.025 |
| Sweden | 0.019 | 0.000 | 3.631 | 0.006 |
| Malta | 0.008 | 0.001 | 10.424 | 0.048 |
| Norway | 0.008 | 0.000 | 0.733 | 0.005 |
| United Kingdom | 0.341 | 0.000 | 26.146 | 0.016 |
| Spain | 0.281 | 0.000 | 1.376 | 0.016 |
| Switzerland | 0.039 | 0.000 | 13.135 | 0.011 |
| Argentina | 0.653 | 0.001 | 4.761 | 0.052 |
| Chile | 0.118 | 0.001 | 3.905 | 0.021 |
| United States of America | 1.580 | 0.000 | 61.193 | 0.015 |
| Canada | 0.169 | 0.000 | 0.069 | 0.014 |
| Uruguay | 0.099 | 0.002 | 6.563 | 0.100 |
| Antigua and Barbuda | 0.002 | 0.002 | 0.310 | 0.062 |
| Dominica | 0.002 | 0.002 | 0.335 | 0.094 |
| Bahamas | 0.014 | 0.003 | 20.310 | 0.132 |
| Belize | 0.008 | 0.002 | 0.596 | 0.083 |
| Barbados | 0.014 | 0.003 | 0.074 | 0.125 |
| Cuba | 0.568 | 0.003 | 0.493 | 0.125 |
| Dominican Republic | 0.313 | 0.003 | 13.352 | 0.119 |
| Grenada | 0.004 | 0.003 | 0.617 | 0.137 |
| Haiti | 0.410 | 0.004 | 18.151 | 0.161 |
| Guyana | 0.015 | 0.002 | 1.117 | 0.080 |
| Jamaica | 0.081 | 0.003 | 3.179 | 0.103 |
| Saint Vincent and the Grenadines | 0.005 | 0.003 | 0.162 | 0.140 |
| Trinidad and Tobago | 0.047 | 0.003 | 0.339 | 0.107 |
| Saint Lucia | 0.008 | 0.004 | 0.185 | 0.151 |
| Bolivia (Plurinational State of) | 0.154 | 0.001 | 1.830 | 0.056 |
| Suriname | 0.028 | 0.004 | 6.382 | 0.174 |
| Peru | 0.258 | 0.001 | 5.594 | 0.028 |
| Colombia | 0.652 | 0.001 | 10.542 | 0.050 |
| Ecuador | 0.135 | 0.001 | 26.927 | 0.031 |
| El Salvador | 0.100 | 0.002 | 5.119 | 0.069 |
| Costa Rica | 0.120 | 0.002 | 4.895 | 0.091 |
| Honduras | 0.124 | 0.002 | 4.283 | 0.061 |
| Guatemala | 0.197 | 0.001 | 8.743 | 0.062 |
| Mexico | 1.399 | 0.001 | 3.276 | 0.042 |
| Panama | 0.074 | 0.002 | 2.991 | 0.068 |
| Paraguay | 0.102 | 0.002 | 182.658 | 0.064 |
| Nicaragua | 0.077 | 0.001 | 4.446 | 0.052 |
| Venezuela (Bolivarian Republic of) | 0.599 | 0.002 | 24.198 | 0.081 |
| Brazil | 4.207 | 0.002 | 2.008 | 0.072 |
| Bahrain | 0.044 | 0.002 | 14.395 | 0.103 |
| Iran (Islamic Republic of) | 0.570 | 0.001 | 168.036 | 0.025 |
| Jordan | 0.228 | 0.002 | 3.310 | 0.087 |
| Algeria | 3.717 | 0.008 | 25.671 | 0.360 |
| Iraq | 0.574 | 0.002 | 4.611 | 0.068 |
| Egypt | 0.323 | 0.000 | 10.599 | 0.015 |
| Kuwait | 0.070 | 0.001 | 2.788 | 0.052 |
| Lebanon | 0.105 | 0.002 | 42.482 | 0.075 |
| Libya | 0.933 | 0.011 | 100.267 | 0.500 |
| Palestine | 0.032 | 0.001 | 1.976 | 0.034 |
| Morocco | 1.557 | 0.006 | 58.345 | 0.252 |
| Qatar | 2.420 | 0.001 | 1.459 | 0.051 |
| Oman | 0.040 | 0.001 | 111.448 | 0.053 |
| Syrian Arab Republic | 0.059 | 0.000 | 15.624 | 0.018 |
| Saudi Arabia | 0.067 | 0.005 | 2.773 | 0.224 |
| Yemen | 2.361 | 0.001 | 67.753 | 0.048 |
| Turkey | 0.257 | 0.003 | 11.757 | 0.131 |
| Tunisia | 2.974 | 0.011 | 126.305 | 0.489 |
| United Arab Emirates | 0.334 | 0.003 | 18.996 | 0.110 |
| Bangladesh | 10.682 | 0.007 | 473.449 | 0.288 |
| Afghanistan | 0.395 | 0.002 | 1.811 | 0.087 |
| Bhutan | 0.040 | 0.005 | 3336.343 | 0.227 |
| Nepal | 75.706 | 0.005 | 59.879 | 0.207 |
| India | 1.328 | 0.005 | 838.518 | 0.229 |
| Angola | 0.306 | 0.002 | 14.395 | 0.066 |
| Congo | 0.077 | 0.002 | 2.806 | 0.074 |
| Pakistan | 17.746 | 0.009 | 3.570 | 0.414 |
| Central African Republic | 0.061 | 0.002 | 0.688 | 0.069 |
| Equatorial Guinea | 0.014 | 0.001 | 38.674 | 0.062 |
| Comoros | 0.039 | 0.006 | 0.840 | 0.260 |
| Democratic Republic of the Congo | 0.833 | 0.001 | 27.968 | 0.060 |
| Eritrea | 0.469 | 0.009 | 2.723 | 0.413 |
| Gabon | 0.019 | 0.001 | 1.810 | 0.054 |
| Burundi | 0.581 | 0.007 | 22.333 | 0.301 |
| Djibouti | 0.059 | 0.005 | 235.375 | 0.225 |
| Ethiopia | 4.835 | 0.007 | 17.937 | 0.294 |
| Malawi | 0.373 | 0.003 | 243.336 | 0.132 |
| Kenya | 5.177 | 0.014 | 3.573 | 0.614 |
| Madagascar | 1.476 | 0.007 | 71.696 | 0.326 |
| Mauritius | 0.087 | 0.005 | 3.948 | 0.225 |
| Rwanda | 0.593 | 0.006 | 28.101 | 0.260 |
| Mozambique | 0.088 | 0.001 | 0.546 | 0.021 |
| Seychelles | 0.013 | 0.010 | 160.814 | 0.424 |
| Somalia | 0.810 | 0.007 | 39.121 | 0.288 |
| Uganda | 4.101 | 0.016 | 199.710 | 0.713 |
| United Republic of Tanzania | 3.395 | 0.008 | 98.395 | 0.373 |
| Zambia | 2.035 | 0.016 | 1.725 | 0.699 |
| Botswana | 0.036 | 0.002 | 1.939 | 0.070 |
| Lesotho | 0.041 | 0.003 | 1.766 | 0.119 |
| Eswatini | 0.021 | 0.002 | 30.205 | 0.101 |
| Namibia | 0.036 | 0.002 | 1.010 | 0.079 |
| Zimbabwe | 0.387 | 0.003 | 19.571 | 0.151 |
| South Africa | 0.654 | 0.001 | 5.532 | 0.050 |
| Burkina Faso | 0.160 | 0.001 | 7.847 | 0.050 |
| Benin | 0.112 | 0.001 | 25.854 | 0.059 |
| Cabo Verde | 0.008 | 0.001 | 15.989 | 0.063 |
| Chad | 0.082 | 0.001 | 0.371 | 0.038 |
| Côte d'Ivoire | 0.389 | 0.002 | 4.042 | 0.086 |
| Cameroon | 0.324 | 0.001 | 18.707 | 0.067 |
| Ghana | 0.022 | 0.000 | 0.787 | 0.004 |
| Guinea-Bissau | 0.021 | 0.001 | 1.106 | 0.069 |
| Gambia | 0.016 | 0.001 | 5.942 | 0.045 |
| Guinea | 0.123 | 0.001 | 1.063 | 0.064 |
| Mali | 0.101 | 0.001 | 2.681 | 0.032 |
| Liberia | 0.054 | 0.001 | 4.861 | 0.058 |
| Mauritania | 0.019 | 0.001 | 0.913 | 0.028 |
| Nigeria | 6.378 | 0.004 | 0.011 | 0.188 |
| Senegal | 0.101 | 0.001 | 5.747 | 0.041 |
| Niger | 0.116 | 0.001 | 4.944 | 0.040 |
| Sao Tome and Principe | 0.000 | 0.000 | 306.446 | 0.006 |
| Togo | 0.122 | 0.002 | 2.431 | 0.085 |
| Sierra Leone | 0.049 | 0.001 | 5.906 | 0.036 |
| Bermuda | 0.002 | 0.002 | 0.198 | 0.087 |
| Greenland | 0.004 | 0.006 | 0.008 | 0.231 |
| American Samoa | 0.005 | 0.009 | 0.080 | 0.364 |
| Monaco | 0.000 | 0.000 | 0.161 | 0.015 |
| Cook Islands | 0.000 | 0.001 | 0.513 | 0.039 |
| Niue | 0.000 | 0.003 | 0.020 | 0.144 |
| Guam | 0.013 | 0.007 | 0.007 | 0.282 |
| Palau | 0.000 | 0.000 | 0.003 | 0.008 |
| Saint Kitts and Nevis | 0.003 | 0.003 | 0.214 | 0.129 |
| Nauru | 0.000 | 0.005 | 0.002 | 0.220 |
| Northern Mariana Islands | 0.005 | 0.008 | 0.101 | 0.330 |
| Puerto Rico | 0.015 | 0.000 | 0.541 | 0.013 |
| San Marino | 0.000 | 0.001 | 0.012 | 0.028 |
| Tuvalu | 0.001 | 0.004 | 0.002 | 0.184 |
| South Sudan | 0.369 | 0.006 | 0.021 | 0.250 |
| Tokelau | 0.000 | 0.004 | 0.119 | 0.158 |
| Sudan | 0.468 | 0.001 | 17.069 | 0.064 |
| United States Virgin Islands | 0.003 | 0.003 | 22.180 | 0.127 |

Table S2 The APC and AAPC of ASMR, ASDR for nasopharynx cancer attributable to occupational formaldehyde from 1990 to 2021.

| Range | Deaths APC (95% CI) | P | Range | DALYs APC (95% CI) | P |
| --- | --- | --- | --- | --- | --- |
| 1990-1993 | 0.7235 (0.2155 to 1.2341) | 0.008 | 1990-1993 | 0.5976 (0.0722 to 1.1258) | 0.028 |
| 1993-2000 | -1.1339 (-1.2947 to -0.9729) | <0.001 | 1993-2000 | -1.2316 (-1.3970 to -1.0658) | <0.001 |
| 2000-2006 | -4.0758 (-4.2822 to -3.8690) | <0.001 | 2000-2006 | -4.1233 (-4.3354 to -3.9107) | <0.001 |
| 2006-2014 | -1.2825 (-1.4158 to -1.1490) | <0.001 | 2006-2013 | -1.5766 (-1.7448 to -1.4081) | <0.001 |
| 2014-2021 | 0.3051 (0.1637 to 0.4468) | 0.003 | 2013-2021 | 0.1562 (0.0379 to 0.2746) | 0.013 |
| Range | Deaths AAPC (95% CI) | P | Range | DALYs AAPC (95% CI) | P |
| 1990-2021 | -1.2496 (-1.3305 to -1.1686) | <0.001 | 1990-1999 | -1.3466 (-1.4302 to -1.2629) | <0.001 |
